# Model for Predicting the Tortuosity of Transport Paths in Cement-Based Materials

**DOI:** 10.3390/ma12213623

**Published:** 2019-11-04

**Authors:** Tongning Cao, Lijuan Zhang, Guowen Sun, Caihui Wang, Ying Zhang, Na Yan, Aoxue Xu

**Affiliations:** 1 School of Materials Science and Engineering, Shijiazhuang Tiedao University, Shijiazhuang 050043, China; 2 Foundational subjects department, Shijiazhuang TieDao University SiFang College, Shijiazhuang 051132, China; 3 Key Laboratory of Smart Material and Structures Mechanics, Hebei Province, Shijiazhuang Tiedao University, Shijiazhuang 050043, China; 4 School of Foreign Studies, Hebei Normal University, Shijiazhuang 050024, China

**Keywords:** pebble, gravel, tortuosity model, morphology characterization, cement-based materials

## Abstract

The tortuosity of the pore structure is an important factor affecting medium (water and harmful ions) transport in cement-based materials. In this study, a new tortuosity model was established to reveal the effect of aggregate size, morphology, and graded media on the transport path in cement-based materials. Based on the stereological principle and the geometric algorithm, the distribution model of the ideal pebble and polygonal aggregate in cement-based materials was given first. Then, based on the image processing technology and MATLAB software, the morphology of the actual aggregate was also characterized to prove the similarity relationship between the ideal aggregate and actual aggregate. The reliability of the tortuosity model was verified by the mercury intrusion porosimetry test and data from other literature. Based on the tortuosity model, the influences of the aggregate particle shape parameters, hydration degree, and water-to-cement ratio on the tortuosity of the transport path were analyzed. Finally, the tortuosity model was further simplified to facilitate engineering application.

## 1. Introduction

The tortuosity in pore structure is one of the key factors affecting the transport performance of cement-based materials. It is usually defined as the ratio of the average diffusion path of a particle to the shortest distance of its straight line [1]. Many scholars have been devoted to testing pore tortuosity through experimental methods, however, it is relatively difficult to test directly. At present, the prediction of pore tortuosity is based on experimental statistical methods or empirical models and the reliability of the model is indirectly proven by experiments. Zhong et al. [2] established a model of pore tortuosity based on the average pore size measured by experiments. De Larrard et al. [3] established the pore tortuosity model based on the theory of an artificial neural network by using the existing experimental data as input data, combined with the numerical model. Piet et al. [4] established a tortuosity model based on the shape and gradation of spherical aggregate particles. The above models were obtained through the experimental statistical method, and the influence of the change of the aggregate’s own morphology on the transport path in cement-based materials was not fully considered, resulting in a certain under estimation between the theoretical prediction and the actual medium transport.

Alireza et al. [5] digitized the geometry and morphology of cracks by image analysis technology, and then established the tortuosity model of cracks. Akira et al. [6] used the three-dimensional spatial distribution model of calcium silicate hydrate (C–S–H) to establish the tortuosity model. Promentilla et al. [7] characterized the three dimensional pore structure based on x-ray synchrotron radiation micro-tomography technology, and established the pore tortuosity model. Zuo et al. [1] established a two-dimensional geometric model of the tortuosity of the transport path by analyzing an image of cement hardened paste by scanning electron microscopy. Liu et al. [8] and Zhou et al. [9] used fractal theory to analyze the tortuosity of capillary pore in cementitious materials, and established the corresponding tortuosity model. Li et al. [10] used macro and micro models to establish the tortuosity model of nano-channels in concrete. Liu et al. [11] provided a multi-scale transport tortuosity model ranging from the nano-scale through to the micro-scale and macro-scale of concrete, which was based on the porous media mechanics and multi-scale method, combined with numerical calculation and 3D visualization. 

However, the above models were obtained by the image analysis method, and the influence of the randomness of aggregate distribution in concrete on the transport path of cement-based materials was not fully considered, resulting in a certain gap between the theoretical prediction and actual medium transport. In this study, based on the stereological principle and geometric method, tortuosity models of cement-based materials containing ideal pebbles and gravel polygonal aggregates were proposed, considering the morphology and gradation of aggregate. Second, the morphology of the actual aggregate was quantitatively characterized by image processing technology considering the effect of actual aggregate morphology on tortuosity, and finally the tortuosity model was validated by the mercury intrusion porosimetry test and the data reported in previous studies.

## 2. Theoretical Model of Tortuosity for Ideal Aggregate in Cement-Based

**Figure 1:**
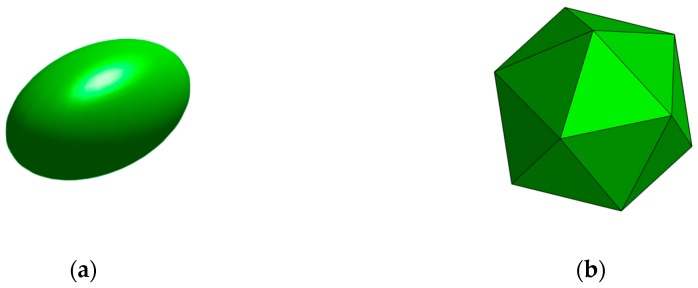
Aggregate shape. (**a**) Pebble aggregate; (**b**) gravel aggregate.

**Figure 2:**
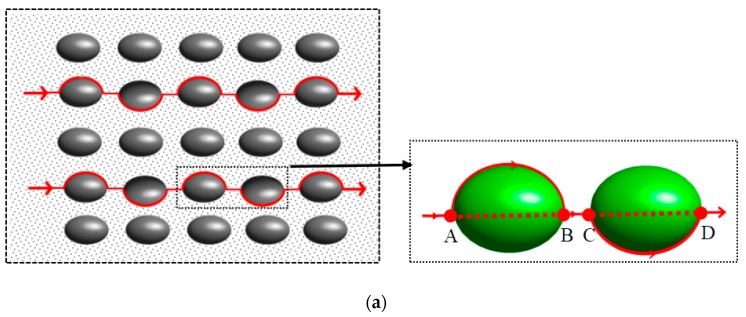

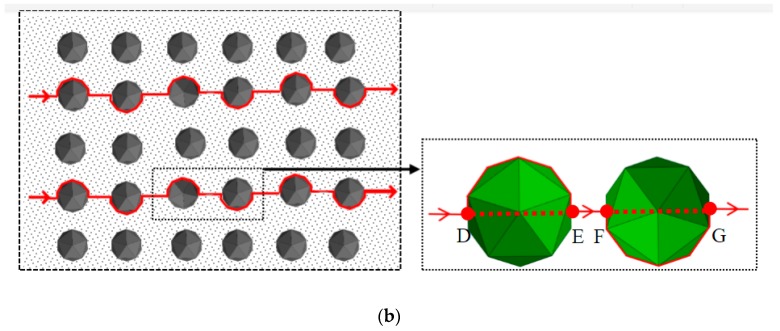
Definition of tortuosity. (**a**) Pebble aggregate; (**b**) gravel aggregate.

In this paper, mortar can be seen as the result of embedding fine aggregates into a cement slurry, and concrete can be seen as the result of embedding coarse aggregates into mortar. Due to the different sizes of the coarse and fine aggregates, we took the grading of the aggregate size as a weight function to homogenize the whole aggregate to obtain the average size of the aggregate. Then, the average size aggregate with the same size was uniformly embedded in the matrix, and the mortar and concrete model after the aggregate homogenization was obtained. The shape of the aggregate varies, as the fine aggregates are small, it is regarded as sphere; coarse aggregates are mainly divided into pebbles and gravel according to different sources, so it is regarded as ellipsoid and polyhedron, respectively [12], as shown in Figure 1. As the aggregate of the same particle size is evenly distributed in the matrix, the representative volume unit was taken, as shown in Figure 2. 

For the pebble aggregate, the red solid line between points AB and CD is expressed in $\bar{s}$, which is the average actual path length of the particles along the aggregate. The red dotted line between points AB and CD is expressed in $\bar{l}$, which is the average displacement length between the start point and the end point. BC is the average distance between the pebble aggregates, expressed in o. For crushed stone aggregates, the red solid line between points DE and FG is expressed in $\bar{s}$, which is the average actual path length of the particles along the aggregate. The red dotted line between point DE and FG is expressed in $\bar{l}$, which is the average displacement length between the starting point and the end point. EF is the average distance between the crushed stone aggregates, expressed in $\bar{\lambda }$. The average tortuosity is defined as the ratio of the actual path length of particles to the displacement length between the starting point and the end point of the particles. Combined with the literature [1], the specific expression of average tortuosity is shown in Equation (1):

(1a) $$\tau_{c}=\frac{{\bar{s}}_{st}+\bar{\lambda_{st}}}{{\bar{l}}_{st}+\bar{\lambda_{st}}}\cdot \frac{\bar{s_{sa}}+\bar{\lambda_{sa}}}{{\bar{l}}_{sa}+\bar{\lambda_{sa}}}\tau_{cp}$$

(1b) $$\tau_{cp}=\eta_{c}\left(1-h_{a}\right)\tau_{uh\text{-}cp}+h_{a}{\left(1+7h_{a}\right)}^{w/c-0.35}\eta_{r}\tau_{h\text{-}cp}$$

where $\bar{s_{sa}}$ and $\bar{s_{st}}$ denote the actual transport path length of the particles moving along the sand aggregate surface and the stone aggregate surface, respectively; ${\bar{l}}_{sa}$ and ${\bar{l}}_{st}$ denote the linear distance between the starting point and the end point of the sand and the stone, respectively; $\bar{\lambda_{sa}}$ and $\bar{\lambda_{st}}$ denote the sand and the stone aggregate spacing, respectively; *h_a_* is the degree of hydration of the cement; *τ_uh-cp_* and *τ_h-cp_* are the tortuosity of the stacked cement particles and the tortuosity of the fully hydrated cement particles, respectively; and *φ_uh-cp_* and *φ_h-cp_* are the porosity of the stacked cement particles and the porosity of the fully hydrated cement particles, respectively, with *φ_uh-cp_* = 0.58; *w* is the mass of water; *c* is the mass of cement.

### 2.1. Derivation of Tortuosity Model

As an indispensable and important part of concrete, the aggregate has a great influence on the corrosion resistance of concrete. The model treated aggregate size as an inclusion embedded in the cement slurry matrix. The shape of the aggregate was mainly pebbles and gravel, as shown in Figure 2. 

The pebble aggregate particles are regarded as rotating ellipsoidal particles with different diameters, embedded in the mortar matrix. If the size distribution function *f_d_* of an elliptical aggregate is known, the short half-axis of the revolving ellipsoid is *b* and the long half-axis is *a*, *k* is the ratio of length to diameter of a revolving ellipsoid. When *k* = *a*/*b* > 1, the ellipsoid is prolate; when *k* = *a*/*b* < 1, the ellipsoid is oblate.

The volume (*V*) of the rotating ellipsoid is obtained as follows:

(2) $$V\left(b\right)=\{\begin{array}{cc} \frac{4\pi }{3}kb^{3} & k>1\\ \frac{4\pi }{3}k^{-2}b^{3} & k>1 \end{array}$$

The surface area (*S*) of the rotating ellipsoid is obtained as follows:

(3) $$S\left(b\right)=\{\begin{array}{cc} 4\pi kb^{2} & k>1\\ 4\pi k^{-2}b^{2} & k<1 \end{array}$$

the equivalent diameter of an aspheric particle is defined as the diameter of a sphere, which equals the volume of the aspheric particle. In other words, the particle size distribution of the non-spherical aggregate particles can be correlated with the particle size distribution of the equivalent spherical particles. The equivalent diameter (*D_eq_*), can be expressed as:

(4) $$D_{\mathrm{eq}}=\left\{ \begin{array}{cc} 2bk^{\frac{1}{3}} & k>1\\ 2bk^{-\frac{2}{3}} & k<1 \end{array}\right.$$

The gravel aggregate particles are regarded as regular octahedron and icosahedron. According to geometry and stereoscopic principles, the relationship between the edge length (*a*) and its circumscribed sphere radius (*r_cir_*) can be expressed as:

(5) $$a=\{\begin{array}{cc} \sqrt{2}r_{cir} & \mathrm{octahedron}\\ \frac{\left(\sqrt{5}-1\right)r_{cir}}{2} & \mathrm{icosahedron} \end{array}$$

The volume (*V*) of the regular octahedron and icosahedron are given as follows:

(6) $$V=\{\begin{array}{cc} \frac{\sqrt{2}}{3}a^{3} & \mathrm{octahedron}\\ \frac{15+5\sqrt{5}}{12}a^{3} & \mathrm{icosahedron} \end{array}$$

The surface area (*S*) of the regular octahedron and icosahedron are given as follows:

(7) $$S=\{\begin{array}{cc} 2\sqrt{3}a^{2} & \mathrm{octahedron}\\ 5\sqrt{3}a^{2} & \mathrm{icosahedron} \end{array}$$

For Plato polyhedral aggregate particles, the particle size of the polyhedral aggregate can be correlated with that of the spherical aggregate by means of an equivalent diameter. For a regular octahedron and icosahedron, its equivalent diameter can be expressed as:

(8) $$D_{eq}=\{\begin{array}{cc} {\left(\frac{1}{\pi }\right)}^{\frac{1}{3}}r_{cir} & \mathrm{octahedron}\\ {\left(\frac{4\sqrt{10+2\sqrt{5}}}{\pi }\right)}^{\frac{1}{3}}r_{cir} & \mathrm{icosahedron} \end{array}$$

We mainly discuss two special grades of aggregate: Fuller grade aggregates and equal volume grade aggregates, which represent the upper and lower limits of aggregate size grading in concrete. Therefore, the size distribution function *f_d_*(*x*) of the polyhedral aggregate can be expressed as [13]:

(9) $$f_{d}(x)=\frac{-q}{(D_{\max\;eq}^{-q}-D_{\min\;eq}^{-q})D_{eq}{}^{q+1}}\left\{ \begin{array}{l} q=2.5\to Fuller\\ q=3\to EVF \end{array}\right.$$

The volume fraction (*f_st_*) of aggregate in concrete can be calculated by the concrete mix ratio. When the edge length of concrete blocks is known to be *L*, the number of aggregates (*N*) can be obtained.

(10) $$N=\frac{f_{st}\cdot L^{3}}{\int_{D_{\min eq}}^{D_{\max\mathrm{eq}}}f_{d}\left(x\right)\cdot V\left(x\right)dx}$$

The average value of the surface spacing between adjacent aggregates can be expressed by the mean free path ($\bar{\lambda }$) [14], which is directly affected by the aggregate volume fraction (*f_s_*) and solid specific surface area (*S_V_*), as shown in Equations (11) and (12):

(11) $$S_{V}=\frac{\int_{D_{\min eq}}^{D_{\max\mathrm{eq}}}Nf_{d}(x)S(x)dx}{L^{3}}$$

(12) $$\bar{\lambda }=\frac{4\left(1{-f}_{s}\right)}{S_{V}}$$

The average arc length ($\bar{l}$) and chord length ($\bar{s}$) of the ellipsoidal aggregate are given as follows, respectively.

(13) $$\bar{l}=2\int_{D_{eq\min}}^{D_{eq\max}}f_{d}(x)bdx$$

(14) $$\bar{s}=\frac{\pi }{4}\left[k+1+\sqrt{2\left(k^{2}+1\right)}\right]\cdot \int_{D_{eq\min}}^{D_{eq\max}}f_{d}(x)bdx$$

The average arc and chord length of the regular octahedron and icosahedron aggregate are given as follows, respectively.

(15) $$\bar{l}=\{\begin{array}{cc} \int_{D_{eq\min}}^{D_{eq\max}}2{\left(\frac{1}{\pi }\right)}^{-\frac{1}{3}}\cdot f_{d}(x)xdx & \mathrm{octahedron}\\ \int_{D_{eq\min}}^{D_{eq\max}}2{\left(\frac{4\sqrt{10+2\sqrt{5}}}{\pi }\right)}^{-\frac{1}{3}}\cdot f_{d}(x)xdx & \mathrm{icosahedron} \end{array}$$

(16) $$\bar{s}=\{\begin{array}{cc} \int_{D_{eq\min}}^{D_{eq\max}}2\sqrt{2}\cdot {\left(\frac{1}{\pi }\right)}^{-\frac{1}{3}}\cdot f_{d}(x)xdx & \mathrm{octahedron}\\ \int_{D_{eq\min}}^{D_{eq\max}}\frac{5\left(\sqrt{5}-1\right)}{2}\cdot {\left(\frac{4\sqrt{10+2\sqrt{5}}}{\pi }\right)}^{-\frac{1}{3}}\cdot f_{d}(x)xdx & \mathrm{icosahedron} \end{array}$$

By substituting Equations (13)–(16) into Equation (1a), the tortuosity of cement-based materials can be expressed as follows:

(17) $$\tau_{m}=\frac{\bar{s_{sa}}+\bar{\lambda_{sa}}}{{\bar{l}}_{sa}+\bar{\lambda_{sa}}}\tau_{cp}$$

(18) $$\tau_{c}=\frac{{\bar{s}}_{st}+\bar{\lambda_{st}}}{{\bar{l}}_{st}+\bar{\lambda_{st}}}\tau_{m}$$

where *τ_c_*, *τ_m_*, and *τ_cp_* are the tortuosity of the concrete, mortar, and porosity of cement pastes, respectively, and the expression is given in [1].

Porosity (*φ*) is also an important parameter affecting ion diffusion performance in concrete. The formula of porosity is as follows [15]:

(19) $$\varphi =\mathrm{Max}\left(f_{vc}\frac{w/c-0.39h_{a}}{w/c+0.32},0\right)$$

where *w* is the mass of water; *c* is the mass of cement; and *f_vc_* is the cement volume fraction.

### 2.2. Numerical Simulation of the Influence of Aggregate Morphology and Grades on Tortuosity

**Figure 3:**
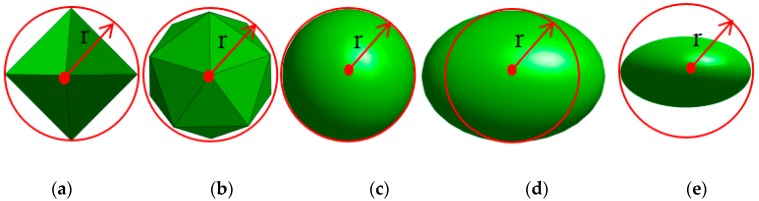
Aggregate shape in the tortuosity model. (**a**) Octahedron; (**b**) Icosahedron; (**c**) Sphere; (**d**) Prolate ellipsoid; (**e**) Oblate ellipsoid.

In theoretical simulation, the aggregate morphology is often regarded as regular octahedron and regular icosahedron, spherical, elliptical, and oblate pebble aggregates, as shown in Figure 3. According to GB/T 14865-2011 [16] in China, the length of pebble particles should be less than 2.4 times the average particle size of the corresponding particle size, and the thickness of pebble particles should be more than 0.4 times the average particle size. Therefore, the range of the length–diameter ratio of the pebble aggregate is 0.4~2.4.

**Figure 4:**
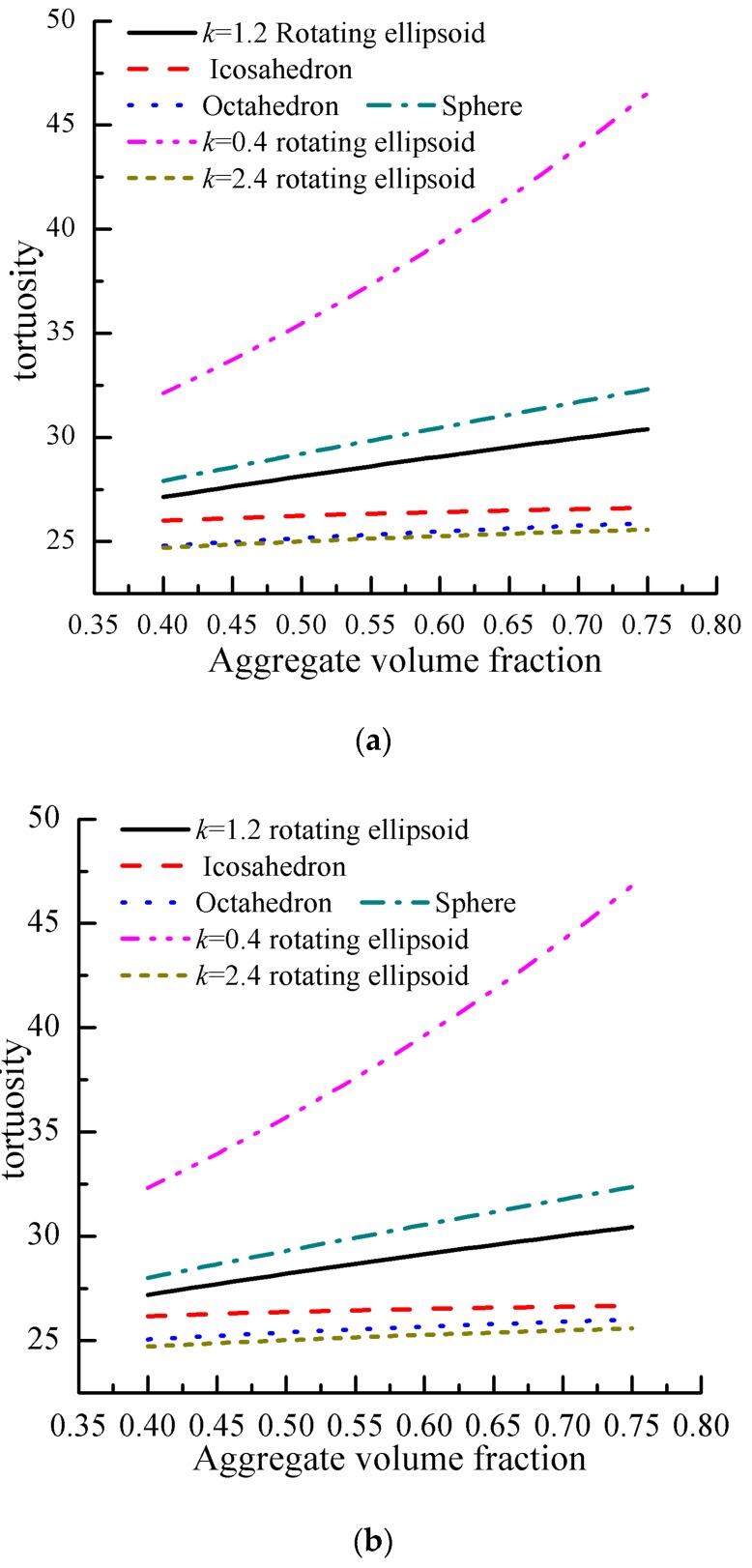
Numerical simulation results of the pore tortuosity. (**a**) Tortuosity under equal volume gradation. (**b**) Tortuosity under Fuller gradation.

According to Equation (17) as established in Section 2.1, for aggregates with Fuller and equal volume distribution, the influence of aggregate morphology including spherical, ellipsoid, octahedron, and icosahedron on tortuosity is shown in Figure 4. It can be seen that the shape, the volume fraction, and the gradation of aggregates are important factors affecting the change in the tortuosity in cement-based materials, and the tortuosity increased with the increase in the aggregate volume fraction.

It can be seen from Figure 4a that when the aggregate gradation and volume fraction were the same, the tortuosity value changes significantly with the morphology of gravel, and its size is sphere > icosahedron > octahedron in turn. Compared with spheres, the tortuosity values of icosahedral and octahedral aggregates were reduced by 11% and 15%, respectively. According to Equations (13)–(16), it is known that the closer the shape the aggregate is to a sphere, the larger the perimeter of the aggregate. With the increase in the perimeter of the aggregate in unit volume concrete, the length of the transport path increases, therefore, the tortuosity value of the concrete increases.

It can also be seen from Figure 4a that when the aggregate gradation and volume fraction are the same, the influence of the pebble morphology on the transport is simulated for the length–diameter ratio *k* = 0.4, 1, 1.2, and 2.4, representing the oblate, spherical, ellipsoid, and needle-bar aggregates. As can be seen from the figure, the tortuosity value was ellipsoid (*k* = 0.4) > sphere (*k* = 1) > ellipsoid (*k* = 1.2) > ellipsoid (*k* = 2.4). Compared with spherical aggregates, the tortuosity value of ellipsoid (*k* = 0.4), ellipsoid (*k* = 1.2), and ellipsoid (*k* = 2.4) increased by 18%, 10%, and 15%, respectively. The tortuosity value decreased with the increase in the length–diameter ratio. The main reason is that when the short axis of the ellipsoidal aggregate is the same as the radius of the spherical aggregate, according to Equations (13) and (14), as the length–diameter ratio increases, the quantity of the aggregate in the unit volume concrete increases, thereby increasing the perimeter of the aggregate and extending the transport path, therefore increasing the tortuosity value. Although the ellipsoidal aggregate in concrete with a length–diameter ratio (*k*) less than 1 has a larger tortuosity value, its compressive strength is lower, so it is used less in engineering.

In addition, from Figure 4a,b, it can be seen that the gradation of the aggregate also has a great influence on the tortuosity values. When the shape and the volume fraction of the aggregates are the same, the tortuosity of cement-based materials with Fuller gradation aggregates is greater than that with an equal volume gradation aggregates. For example, when the rotary ellipsoid and regular icosahedron *k* = 1.2 changed from 0.4 to 0.75, concrete tortuosity and Fuller gradation at the same volume gradation decreased by 7% and 9%, respectively. The equal volume aggregate gradation and Fuller aggregate gradation represent the upper and lower limits of concrete aggregate gradation, respectively. When the aggregate shape and volume fraction are the same, the amount of aggregate under the Fuller gradation in the unit volume concrete will be larger than that of the equal volume gradation. The more aggregates there are in the concrete, the longer the aggregate circumference sum, resulting in a longer ion transport path and the greater the tortuosity.

## 3. Morphological Characterization of Actual Aggregate in Cement-Based Materials

The tortuosity model established in Section 2.1 was mainly based on ideal aggregate particles, which has a certain gap with the actual aggregate morphology. The theoretical prediction in Section 2.2 also confirms that the aggregate morphology has a great influence on the pore tortuosity in cement-based materials. Therefore, it is necessary to carry out statistical characterization of the actual aggregate morphology to establish the similarity relationship between the ideal and actual aggregate. There are many parameters to represent the morphological characteristics of aggregates such as flatness, aspect ratio, micro-characteristics, roundness, practical sphericity, roughness, corner parameters, axial coefficient, roundness, etc. [17,18,19,20,21,22]. According to the parameters required for the tortuosity model established in Section 2.1, the parameters of the micro-characteristics were selected to characterize the morphology of the actual pebble and gravel aggregates.

### 3.1. Expressions of the Microscopic Characteristics of Actual Aggregate Particles

The microscopic characteristics refer to the ratio between the equivalent perimeter (*P_e_*) and the real perimeter (*P*) of the aggregate particles. In this way, the microscopic characteristics of pebbles can be described as the ratio of the perimeter of the equivalent ellipse circumference of particles to the real perimeter of the particles. The gravels are described as the ratio of the perimeter of a regular decagon tangential to the perimeter of a particle with an equal area. 

Thus, the microscopic characteristics (*ξ*) of pebbles and gravel can be expressed as follows:

(20) $$\xi =\frac{P_{e}}{P}$$

Thus, the *ξ* of pebbles can be expressed as follows:

(21) $$\xi =\frac{\frac{\pi }{2}\left[\sqrt{\frac{A}{\pi \sqrt{1-e^{2}}}}+\sqrt{\frac{A\sqrt{1-e^{2}}}{\pi }}+\sqrt{2\left[\frac{A}{\pi \sqrt{1-e^{2}}}+\frac{A\sqrt{1-e^{2}}}{\pi }\right]}\right]}{P}$$

where *A* represents the sum of the pixels in the boundary area of particles; *P* represents the sum of the continuous pixels in the boundary area of particles; *P_e_* represents the circumference of the equivalent ellipse of the object; and *e* represents the eccentricity of the equivalent ellipse.

Gravel is often regarded as octahedrons and icosahedrons, therefore, its projection shape is regular quadrilateral and decagon, respectively. The *ξ* of gravel is as follows:

(22) $$\xi =\{\begin{array}{cc} \frac{2\sqrt{2}D_{eq}}{P} & \mathrm{octahedron}\\ \;\frac{2D_{eq}}{P\cdot \sqrt{10+2\sqrt{5}}} & \mathrm{icosahedron} \end{array}$$

According to Equations (20) and (21), the tortuosity of the actual aggregate concrete can be expressed as:

(23) $$\tau_{c}=\frac{\bar{s_{st}}+\bar{\lambda_{st}}}{\xi_{st}\left(\bar{l_{st}}+\bar{\lambda_{st}}\right)}\tau_{m}$$

(24) $$\tau_{m}=\frac{\bar{s_{sa}}+\bar{\lambda_{sa}}}{\xi_{sa}\left(\bar{l_{sa}}+\bar{\lambda_{sa}}\right)}\tau_{cp}$$

where *ξ_st_* is the microscopic characteristics of sand and *ξ_sa_* is the microscopic characteristics of stone. 

### 3.2. Characterization of Microscopic Properties of Actual Aggregate

**Figure 5:**
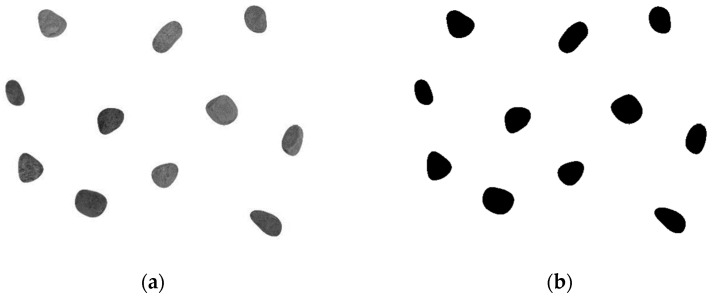

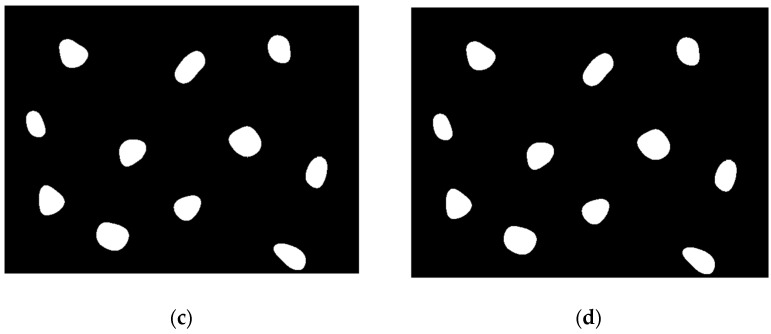
Analysis and calculation process of the aggregate parameters by the MATLAB program. (**a**) Gray image; (**b**) binary image; (**c**) reverse binary image; (**d**) optimized image.

The specific processing method is as follows:

(1) Sampling through the quartile method and laying it on a white background plane to avoid overlapping aggregate particles as far as possible.

(2) The images of coarse and fine aggregate particles are taken by a digital camera with more than 5 million pixels in the JPG file format. According to the JPG file format image obtained above, this paper used MATLAB programming software (MathWorks company of Beijing, China) to process the images of coarse and fine aggregate particles.

(3) The image in the JPG file format taken by digital camera is a RGB image that can be transformed into a gray image, which can greatly reduce the amount of image data and facilitate image processing, as shown in Figure 5a. By analyzing the gray histogram of the image to determine the optimal threshold needed for image segmentation, the threshold is used for binarization, and the gray image is transformed into a binary image, reverse binary image, and optimized image, as shown in Figure 5b–d.

(4) Then, the image area, edge circumference, eccentricity of the equivalent ellipse, the long axis of the equivalent ellipse, the short axis of the equivalent ellipse, and the diameter of the equivalent circle are extracted.

(5) Finally, the shape parameters of the aggregates are calculated by the weighted average method:

(25) $$F=\frac{\sum f_{s}\xi }{100}$$

In the formula, *F* represents the shape parameters of the aggregate particles as a whole; *f_s_* represents the volume fraction of the aggregates per grain size; and *ξ* represents the shape parameter value of the corresponding granular aggregate.

### 3.3. Testing Process and Statistical Results of Micro-Properties of Fine Aggregate

**Figure 6:**
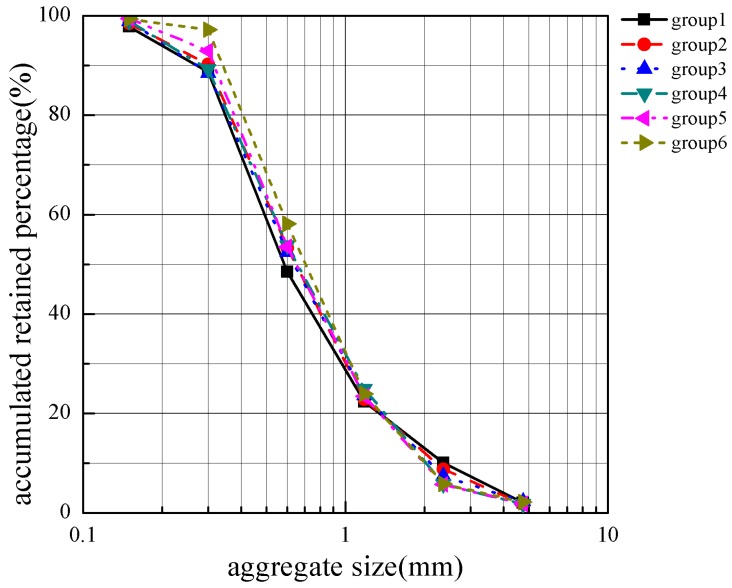
Sieving results of the sample.

After the obtained sand sample was uniformly stirred, the sample was divided by the quarter method, and after washing with water, six samples of not less than 550 g each were taken. The two samples were respectively placed in an oven at a temperature of 105 ± 5 °C and dried to a constant weight, and then 500 g of the sample was taken. The sieving results of the sample are shown in Figure 6.

According to the definition of the micro-characteristics and considering the actual situation, sand grains below 1.18 mm were regarded as spheres and their shape parameters were 1, where the sand particle size in the range of 1.18~2.36 mm and 2.36~4.75 mm were counted by the MATLAB programming language, according to the image processing steps given in Section 3.2. The statistical results of the micro-characteristic are shown in Table 1 and Table 2, respectively. It can be seen that the micro-characteristics of sand particles with particle sizes of 1.18~2.36 mm and 2.36~4.75 mm were between 0.95~0.98.

### 3.4. Testing Process and Statistical Result of Micro-Properties of Coarse Aggregate

**Figure 7:**
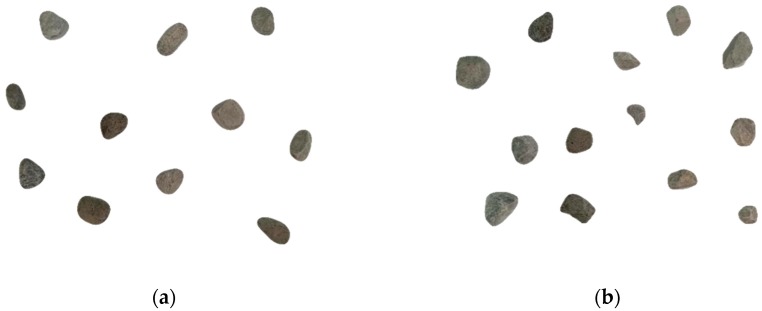
Actual aggregate morphology. (**a**) Actual pebble aggregate; (**b**) actual gravel aggregate.

**Figure 8:**
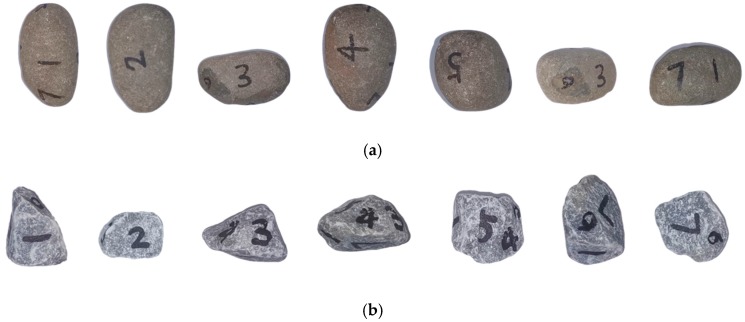
Aggregate projection shapes at different angles. (**a**) Pebble; (**b**) gravel.

The coarse aggregate was divided by a quartering method to 22 kg, and then pebble and gravel aggregate samples with particle sizes of 5~10 mm and 10~20 mm were randomly sampled as shown in Figure 7. For the image statistics, each aggregate sample was photographed from seven angles as shown in Figure 8. After image processing with MATLAB, the statistical results of the morphological parameters of pebbles and gravels are shown in Table 3, Table 4, Table 5, Table 6 and Table 7, respectively.

Table 3, Table 4, Table 5, Table 6 and Table 7 present the statistical morphological parameters of each aggregate including the area, circumference, eccentricity of equivalent ellipse, long and short axes, and equivalent diameter of circle by using MATLAB programming software from seven angles. These statistical morphological parameters were brought into Equation (22) and the average value of the shape parameters of the weighted aggregates were calculated and are given in Table 8. As can be seen from Table 8, the shape parameters of the aggregate with particle sizes of 5~10 mm and 10~20 mm ranged from 0.89~0.95.

## 4. Verification of Tortuosity Model

Due to the complexity of pore tortuosity, it is relatively difficult to measure directly. At present, mercury intrusion porosimetry (MIP) is commonly used to characterize the pore structure characteristics of cement-based materials such as porosity, tortuosity, and pore connectivity in cement-based materials [23]. Therefore, it was introduced in this paper to verify the reliability of the model established.

### 4.1. Preparation of Samples

Considering the possible errors caused by coarse aggregates in concrete, mortar specimens were selected to validate the model, where the water -o-cement ratio was 0.45, and the volume fraction of sand was 20%, 40%, and 50%, respectively. The gradation of sand is shown in Figure 6. The stirred slurry was first poured into a plastic beaker of 500 mL, shaken for 3 min, and then injected into a PVC (Polyvinyl chloride) tube with a diameter of 16 mm, and shaken for another 2 min. The purpose of vibration was to eliminate the bubbles produce d during mixing to reduce the test error. After three days of curing in the standard room, the sample was taken out from the PVC tube and then both ends were removed. The intermediate sample was taken to a height of about 15 mm and then standardized to the specified age for testing the porosity tortuosity. The sample was immersed in anhydrous ethanol before the test to prevent hydration.

### 4.2. Testing Process and Results of Pore Tortuosity 

AutoPore IV9500 produced by Micrometrics (Ottawa, Canada) was used for MIP, where the maximum pressure can reach 415 MPa, and the pore size of the measurement range was 3 nm to 360 μm. The measurement was conducted in two stages: a manual low pressure from 0.003 MPa to 0.21 MPa, and automatic high pressure from 0.21 MPa to 242 MPa. After completing the low-pressure test, the penetrometer was taken out from the low-pressure chamber and weighed. The high-pressure test was carried out. The contact angle was set at 130 degrees and the balance time was 30 s. 

The experimental and theoretical prediction results of tortuosity in cement-based materials are shown in Table 9, where the micro-characteristics of 2.36~4.75 mm particle size are 0.96. By substituting it into Equations (19)–(22), the theoretical results of actual tortuosity can be obtained. As can be seen from Table 9, the theoretical prediction results coincide with that of the MIP test, whose maximum error is 10.41%.

To further verify the reliability of the tortuosity model, the tortuosity value given by Zuo [1] was compared with the predicted results, as shown in Table 10. It can be seen from the table that the relative error between the measured and predicted values was less than 10%, which also prove that the prediction model of tortuosity and the characterization of morphological parameters are reasonable.

## 5. Sensitivity Analysis of Model Parameters

### 5.1. Effect of Aggregate Morphology on Tortuosity

**Figure 9:**
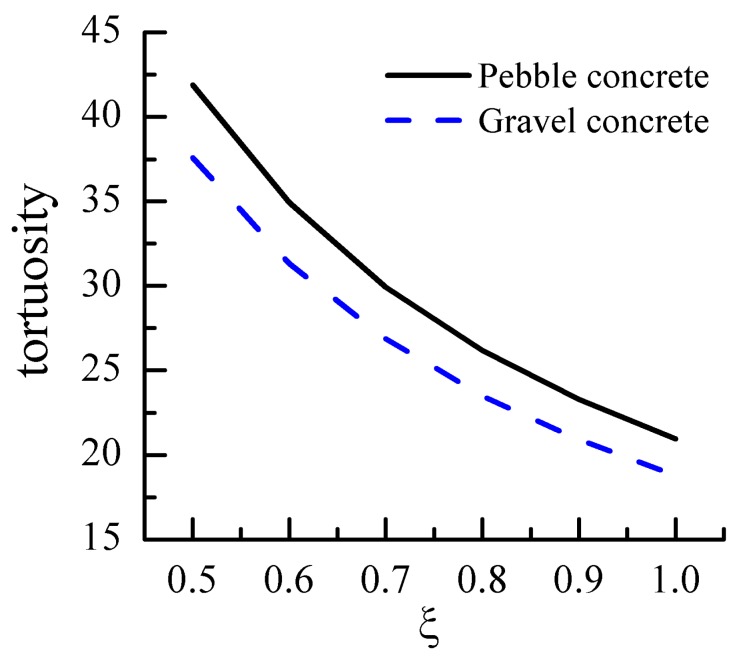
Effect of aggregate particle shape parameters on concrete tortuosity.

Aggregate morphology is mainly expressed by the microscopic characteristics of aggregate particles (ξ), where we assumed that ξ = 0.5–1 and was brought into Equation (22). The relationship of the ξ and tortuosity is demonstrated in Figure 9. It can be seen that the tortuosity values of cement-based with pebble and gravel aggregates decreased with the increase in the ξ. For example, the tortuosity values at *ξ* = 0.5 decreased by 51% and 50% with respect to that at *ξ* = 1.0 for the pebble and gravel aggregate, respectively. The main reason is that the *ξ* values affect the roughness of the aggregate surface. The smaller the *ξ* values, the rougher the surface and the longer the perimeter of the aggregate, which increase the transport paths of the medium and are conducive to enhancing the durability of cement-based materials.

### 5.2. Effect of Hydration Degree on Tortuosity

**Figure 10:**
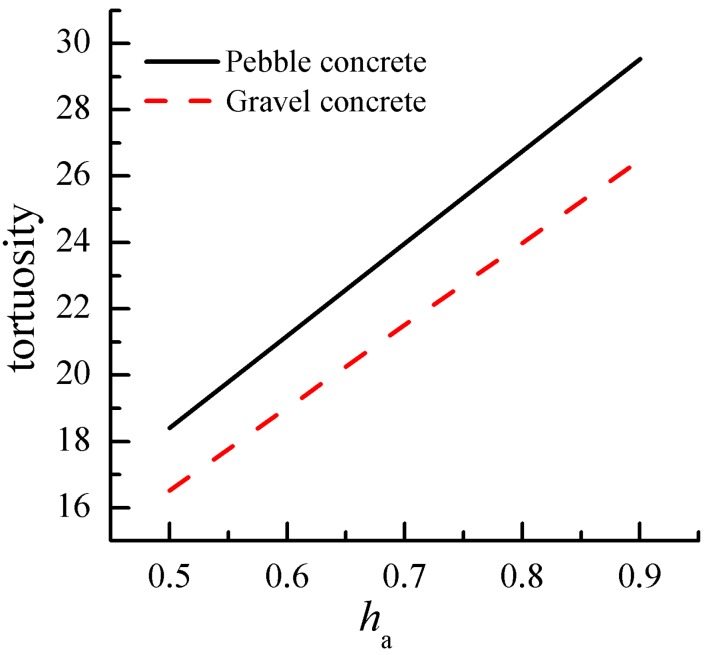
Effect of hydration degree on concrete tortuosity.

The degree of hydration of cement (*h_a_*) has a great influence on the pore tortuosity of cement paste, where we assumed that *h_a_* = 0.5~0.9 and was brought into Equation (1b). The relationship of the *h_a_* and tortuosity is demonstrated in Figure 10. It can be seen that the tortuosity values of cement-based with pebble and gravel aggregates increased with the increase in the *ξ*. For example, the tortuosity values at *h_a_* = 0.5 decreased by 37.6% and 37.5% with respect to that at *h_a_* = 0.9 for the pebble and gravel aggregate, respectively. The main reason is the decrease in porosity and pore connectivity with the increase in hydration degree of cement, which leads to the increase in tortuosity.

### 5.3. Effect of Water–Cement Ratio on Transport

**Figure 11:**
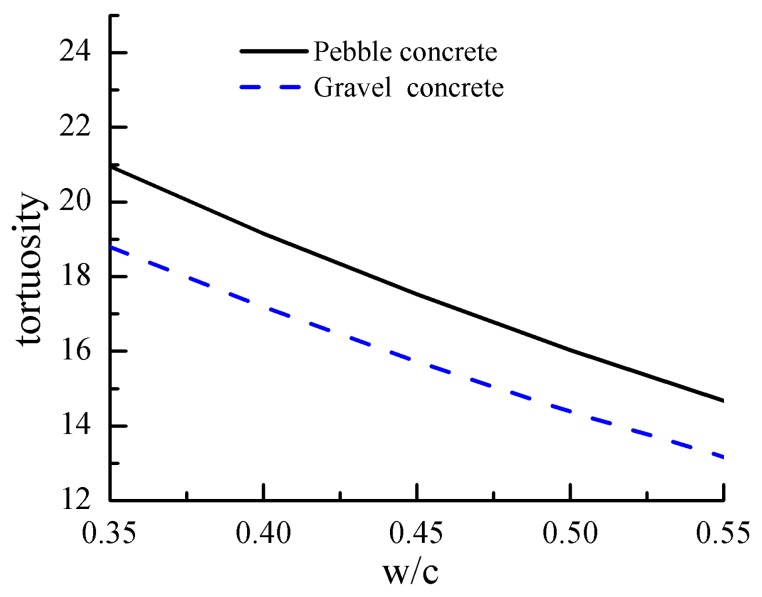
Effect of water-to-binder ratio on the tortuosity of concrete.

Water–cement ratio (w/c) has a great influence on the pore structure of cement matrix between the aggregate and matrix, where we assumed that w/c = 0.35~0.55 and was brought into Equation (1b). The relationship of the w/c and tortuosity is demonstrated in Figure 11. It can be seen that the tortuosity values of cement-based with pebble and gravel aggregates decreased with the increase in the *ξ*. For example, the tortuosity values at w/c = 0.35 decreased by 29.9% and 30% with respect to that at w/c = 0.55 for the pebble and gravel aggregate, respectively. The main reason is the decrease in porosity and pore connectivity with the increase in the hydration degree of cement, which leads to the increase in tortuosity.

## 6. Simplified Application of Tortuosity Model in Engineering

In practical engineering, the pore tortuosity is an important parameter for predicting the transport of medium in structural concrete. The tortuosity model established above fully considers many factors such as the morphology of the aggregate, volume fraction of the aggregate, aggregate gradation and hydration degree of the slurry, etc. There are many parameters and complex calculation processes, but in practical engineering, there is an urgent need for a simpler tortuosity model in the process. Therefore, the above-mentioned tortuosity model can be simplified, and its expression can be expressed as follows:

In practical engineering, it is necessary to simplify the tortuosity formula to facilitate the practical application of engineering. Table 11 is calculated according to Equation (1b). In Table 12, t_1_ represents the ratio of the circumference of the maximum section of the sphere, the ellipsoid of rotation, the regular octahedron, and the regular icosahedron to the circumference of the circumferential circle. t_2_ represents the ratio of the area of the maximum section of sphere, ellipsoid of rotation, regular octahedron, and regular icosahedron to the area of the circumscribed circle. t_3_ represents the ratio of the volume of sphere, ellipsoid of rotation, regular octahedron, and regular icosahedron to the volume of the circumscribed sphere.

The assumptions were as follows:

(1) The shape of the fine aggregate is a spherical shape; the shape of the coarse aggregate is a spheroidal shape and a regular polyhedron;

(2) Through the screening experiment, the grading of coarse and fine aggregates was statistically calculated, and the aggregate was weighted according to the grading and screening particle size to obtain the average particle size of the aggregate $\bar{D_{s}}$.

The average particle size of the fine aggregate is $\bar{D_{sa}}$. The average particle size of the coarse aggregate is $\bar{D_{st}}$. R is the radius of the average particle size.

(1) The volume fraction of the aggregate *f_s_* is calculated by the concrete mix ratio.

(2) The volume fraction of fine aggregate is *f_sa_*. The volume fraction of the coarse aggregate is *f_st_*;

(3) In order to simplify the calculation, the regular polyhedron is unified into a rotating ellipsoid in combination with Table 12.

By introducing Equations (26) and (27) into (23) and (24), the simplified tortuosity formula can be expressed as follows in Equation (28):

(26a) $$\lambda =\frac{4\left(1{-f}_{s}\right)}{S_{V}}$$

(26b) $$S_{V}=\frac{Nt_{2}4k\pi \bar{R^{2}}}{L^{3}}$$

(26c) $$N=\frac{4L^{3}f_{s}}{3\pi kt_{3}\bar{R^{3}}}$$

(27) $$s=t_{1}2k\pi D$$

(28) $$\tau_{c}=\frac{t_{1}\bar{D_{st}}+\frac{2\left(1-f_{st}\right)t_{3}\bar{D_{st}}}{3f_{st}t_{2}}}{\left(\bar{D_{st}}+\frac{2\left(1-f_{st}\right)t_{3}\bar{D_{st}}}{3f_{st}t_{2}}\right)}\cdot \frac{\pi \bar{d}+\frac{2\left(1-f_{sa}\right)\bar{D_{sa}}}{3f_{sa}}}{\left(\bar{d}+\frac{2\left(1-f_{sa}\right)\bar{D_{sa}}}{3f_{sa}}\right)}\tau_{cp}$$

## 7. Conclusions

(1) Based on the stereological principle, the tortuosity model of ideal pebble and gravel aggregates in cement-based materials was established by the geometric method. Then, the microscopic characteristics of the actual sand and coarse aggregate were counted by using the image processing software MATLAB. The results show that the microscopic characteristics of particle size were 0.95, 0.96, 0.93, and 0.92 in the range of 2.36–4.75 mm, 5–10 mm, and 10–20 mm, respectively. On this basis, the prediction model of the tortuosity degree of actual aggregate in the actual cement-based materials was established. The reliability of the tortuosity model was proven by the mercury intrusion test data and experimental data reported in the literature. The maximum error of the model was 8.59%.

(2) The effects of the aggregate particle shape parameters, aggregate volume fraction, and water cement ratio on the tortuosity of the transport path in concrete were analyzed. The tortuosity decreased with the increase in the aggregate particle shape parameters, increased with the increase in the cement hydration degree, and increased with the increase in the water cement ratio. Combined with engineering practice and considering the morphological characteristics of the aggregate, a simplified tortuosity model was presented, which facilitates engineering applications.

## Figures and Tables

**Table 1:** Statistical results of sand particles for 1.18~2.36 mm sizes.

No.	S1 #	S2 #	S3 #	S4 #	S5 #	S6 #
Average value	0.98	0.96	0.95	0.95	0.97	0.98
Standard deviation	0.03	0.04	0.03	0.04	0.03	0.02

**Table 2:** Statistical results of sand particles for 2.36~4.75 mm sizes.

No.	S1 #	S2 #	S3 #	S4 #	S5 #	S6 #
Average value	0.97	0.98	0.95	0.96	0.97	0.96
Standard deviation	0.04	0.03	0.03	0.02	0.04	0.03

**Table 3:** Statistical results of the aggregate from different angles.

Aggregate Shape	Number	Area/px	Perimeter/px	Eccentricity	MajorAxis Length/px	Minor Axis Length/px	Equiv. Diameter/px
pebble	1	1289	133.51	0.77	51.19	32.52	40.51
	2	1814	186.07	0.50	53.47	46.17	48.05
	3	2130	169.04	0.61	59.07	46.68	52.07
	4	1660	152.43	0.64	53.27	40.87	45.97
	5	1271	244.92	0.74	53.89	36.28	40.22
	6	1668	163.38	0.87	66.20	32.84	46.08
	7	1003	140.43	0.89	54.25	24.35	35.73
gravel	1	2124	177.61	0.84	71.25	38.50	52.01
	2	2077	169.94	0.81	67.06	39.72	51.42
	3	1614	181.47	0.79	59.29	36.23	45.33
	4	1478	188.16	0.68	52.06	37.99	43.38
	5	1229	140.76	0.76	49.45	32.17	39.55
	6	1481	152.26	0.74	53.37	35.71	43.42
	7	1338	133.73	0.57	45.73	37.44	41.27

**Table 4:** Statistical results of the pebble aggregate for 5~10 mm particle sizes.

Number	Area/px	Perimeter/px	Eccentricity	MajorAxis Length/px	MajorAxis Length/px	Equiv. Diameter
1	3262	226.27	0.43	68.41	61.85	64.44
2	1725	265.29	0.81	69.16	40.79	46.86
3	2937	201.79	0.66	71.19	53.23	61.15
4	1888	156.86	0.62	55.65	43.84	49.02
5	3375	228.08	0.83	87.96	49.65	65.55
6	2108	178.84	0.74	64.22	43.14	51.80
7	1126	126.66	0.81	50.39	29.36	37.86
8	1518	148.94	0.54	48.83	41.02	43.96
9	1395	143.56	0.61	47.63	37.79	42.14
10	1350	200.77	0.65	48.94	37.32	41.45

**Table 5:** Statistical results of the pebble aggregate for 10~20 mm particle sizes.

Number	Area/px	Perimeter/px	Eccentricity	MajorAxis Length/px	MajorAxis Length/px	Equiv. Diameter
1	60,920	961.32	0.67	323.96	241.61	278.51
2	64,336	1015.77	0.67	334.66	248.11	286.21
3	61,845	1530.84	0.71	338.64	238.64	280.61
4	62,277	1776.39	0.79	386.19	233.72	281.59
5	54,945	1221.35	0.69	268.62	226.81	264.49
6	54,781	975.18	0.76	329.07	214.08	264.10
7	76,626	1403.14	0.74	389.22	262.78	262.35
8	64,833	1719.12	0.71	353.57	250.07	287.26
9	68,552	1485.87	0.51	323.22	277.23	295.44
10	53,980	939.844	0.74	322.72	214.81	262.16
11	66,200	1007.14	0.80	378.02	226.63	290.32
12	56,275	1034.77	0.74	328.29	221.63	267.68
13	78,887	1386.09	0.69	375.78	271.08	266.93
14	68,771	1826.98	0.68	353.69	256.89	295.91

**Table 6:** Statistical results of the gravel aggregate for 5~10 mm particle sizes.

Number	Area/px	Perimeter/px	Eccentricity	MajorAxis Length/px	MajorAxis Length/px	Equiv. Diameter
1	2335	193.77	0.50	59.43	51.64	54.52
2	1242	128.94	0.59	44.84	36.27	39.76
3	1267	165.47	0.93	69.61	24.91	40.16
4	1380	150.43	0.57	46.57	38.36	41.91
5	1590	159.13	0.84	61.83	33.65	44.99
6	2380	251.17	0.84	78.32	42.57	55.04
7	2504	214.60	0.71	67.67	47.80	56.46
8	1342	184.98	0.54	46.06	38.63	41.33
9	2127	225.69	0.83	71.51	39.62	52.04
10	1547	158.88	0.73	54.46	37.10	44.38
11	1688	175.30	0.79	60.87	35.33	46.08

**Table 7:** Analysis results of the gravel aggregate for 10~20 mm particle sizes.

Number	Area/px	Perimeter/px	Eccentricity	MajorAxis Length/px	MajorAxis Length/px	Equiv. Diameter
1	74,768	1419.45	0.71	368.95	261.22	308.54
2	97,995	3081.72	0.64	418.67	321.58	353.23
3	88,353	1863.01	0.85	464.77	247.19	335.40
4	70,326	1515.84	0.83	400.65	224.80	299.25
5	78,879	1556.75	0.81	426.32	249.97	266.91
6	84,365	1292.03	0.79	426.08	256.52	327.75
7	55,138	2146.55	0.76	346.99	223.76	264.96
8	70,002	3779.76	0.76	414.74	266.80	298.54
9	54,135	1629.62	0.67	325.22	240.87	262.54
10	56,826	908.23	0.70	320.44	228.80	268.99
11	52,974	913.38	0.71	266.23	222.47	259.71
12	92,125	1280.87	0.81	454.03	269.28	342.48
13	51,472	2047.56	0.78	340.04	209.88	256.00
14	59,454	1157.89	0.81	367.35	213.16	275.13

**Table 8:** Average statistical results of the aggregate particle shape parameters.

Aggregate Type	Aggregate Size	Shape Parameter
pebble	5–10 mm	0.92
pebble	10–20 mm	0.95
gravel	5–10 mm	0.91
gravel	10–20 mm	0.89

**Table 9:** Comparison of the test and model prediction results.

Number	Measured Average Porosity (%)	Measured Average Tortuosity	Simulated Average Tortuosity	Error (%)
1	19.78	35.71	39.43	10.41
2	15.47	11.83	12.91	9.12
3	12.38	10.51	9.85	6.27

**Table 10:** Comparison of the test results and model results.

Number	Water Cement Ratio	Measured Average Porosity (%)	Measured Average Tortuosity	Simulated Average Tortuosity	Error (%)
SLZ0.35	0.35	10.19	16.11	15.49	3.84
SLZ0.45	0.45	12.84	14.07	12.86	8.59
SLZ0.55	0.55	12.26	14.21	13.55	4.64
SLZ0.65	0.65	10.53	11.72	10.86	7.33

**Table 11:** *τ_cp_* ranges.

w/c	0.3–0.4	0.4–0.5	0.5–0.6
$\tau_{cp}$	17.47–14.37	14.36–11.82	11.82–9.73

**Table 12:** Range of values of the aggregate parameters *t_i_* for different shapes.

Shape	*t*_1_	*t*_2_	*t*_3_
Sphere	1	1	1
Ellipsoid	1~1.77	1~6.52	1~6.52
Octahedron	0.9	0.55	0.32
Icosahedron	0.98	0.76	0.61
